# Preparation and Characterization of New Sol–Gel Hybrid Inulin–TEOS Adsorbent

**DOI:** 10.3390/polym13081295

**Published:** 2021-04-15

**Authors:** Hartina Mohd Yusop, Annur Isma Husna Mohd Ismail, Wan Norfazilah Wan Ismail

**Affiliations:** Faculty of Industrial Sciences and Technology, Universiti Malaysia Pahang, Lebuhraya Tun Razak, Gambang, Kuantan 26300, Pahang, Malaysia; tinaz4510@gmail.com (H.M.Y.); annurisma@gmail.com (A.I.H.M.I.)

**Keywords:** adsorbent, biopolymer–silica hybrid, hybrid sol–gel, sol–gel process

## Abstract

A new biopolymer–silica hybrid material consisting of inulin and tetraethoxysilane (TEOS) for use as an adsorbent was successfully synthesized via the sol–gel method in acidic conditions. The hydrolysis and condensation processes were attained in water/ethanol solution. Three molar ratios of inulin:TEOS (1:1, 1:2, and 2:1) were prepared and dried at various temperatures (50, 60, and 70 °C). The optimized molar ratio of 2:1 with a drying temperature of 70 °C was found to obtain the best morphology and characteristics for absorbent properties. Fourier transform infrared spectroscopy (FTIR) analysis showed a strong interaction between inulin and TEOS, which was also observed using energy dispersive X-ray spectroscopy (EDX). Field emission scanning electron microscopy (FESEM) images revealed the presence of nanoparticles on the rough surface of the hybrid sol–gel. X-ray diffractometer (XRD) analysis showed the amorphous state of the silica network where the inulin existed as an anhydrous crystalline phase. Brunauer–Emmet–Teller (BET) analysis confirmed that the composite was mesoporous, with 17.69 m^2^/g surface area and 34.06 Å pore size. According to thermogravimetric analysis (TGA) results, the hybrid inulin-TEOS adsorbent was thermally stable under a temperature of 200 °C.

## 1. Introduction

Various methods have been employed for wastewater treatment, such as adsorption, ion exchange, precipitation, membrane separation, electrochemical conversion, and biodegradation [1]. Compared to other methods, adsorption techniques are mostly preferred due to their simple operation and implementation, low cost, high performance, efficient regeneration, and eco-friendly operating system [2]. Biopolymer-based hybrid adsorbents based on polysaccharides have gained interest and become significant for wastewater treatment due to their low toxicity, high biodegradability, and wide availability [3]. Chitosan, for example, has received great attention due to its abundant quantity of amino acids and hydroxyl groups, which play crucial roles in sorption processes [4]. However, chitosan shows poor mechanical strength and thermal resistance, weak stability and acid solubility, and low surface area [2]. Furthermore, the use of more than 50% chitosan in biopolymer-based hybrid absorbents could increase the manufacturing cost [5]. Thus, the use of other types of biopolymer should be considered in order to reduce the cost.

Inulin, which is another form of polysaccharide, was proposed as a new formulation in order to develop low-cost biopolymer-based hybrid adsorbent. It is a heterogeneous biopolymer found in various types of plants and comprises a repetitive fructosyl moiety linked by β (2,1) bonds and chain terminating glucosyl moieties, and is considered to be a Generally Recognized as Safe (GRAS) substance. Inulin is relatively dissolvable in water, which allows it to be added to aqueous medium without any precipitation forming [6]. Inulin and its derivatives have been used in a wide range of applications, including pharmaceuticals and water purification, as a flocculant due to its attractive properties. These properties include that it is versatile and biocompatible, a water-soluble carbohydrate, nontoxic, hydrophilic, and biodegradable [7,8].

In addition, inulin and its derivatives have also been used in adsorption applications. Preliminary experiments have been conducted in batch reactors, focusing on the biosorption of copper, zinc, and lead onto chicory pulp containing cellulose, lignin, pectin, and inulin as the main materials. The results show promising adsorption capacity of metal ions between 50 and 100 mg/g [9]. Hernández-Martínez et al. used blended inulin with a polyurethane matrix for the removal of lead ions [6]. Inulin-type fructan binds with activated charcoal, resulting in a spontaneous and exothermic adsorption process [10]. Multifunctional tunable p(inulin) microgels prepared via crosslinking within microemulsion have an adsorption capacity that is influenced by their zeta potential and porosity [11].

The sol–gel technique was used to prepare an inulin-based hybrid adsorbent. This technique involves the hydrolysis and condensation process, which starts from using an alkoxysilane in alcohol and other polar solvents. This process leads to a solution or colloidal suspension of siloxane polymer [12]. Synthesizing with this method produces highly porous and nanocrystalline materials [13]. TEOS, a common silica precursor, was used in this research as it is nontoxic and costs less compared to other types of precursors. Additionally, it can generate derivative compounds by assisting in the reaction of hydroxyl and amine groups [14]. Two parameters that affect the morphology and characterization of inulin–TEOS adsorbent, molar composition and temperature, were optimized.

## 2. Materials and Methods

The precursor TEOS and biopolymer inulin were purchased from Aladdin. Ethanol (EtOH) and hydrochloric acid (HCl) were obtained from Sigma Aldrich and used as co-solvent and catalyst, respectively. Distilled water as solvent was prepared in the laboratory. Sodium hydroxide (NaOH), which was used to prepare the inulin solution, was purchased from R&M Chemicals. The inulin solution was prepared by dissolving 1.0 g of inulin powder into 10 mL of 0.2 M NaOH.

The inulin–TEOS hybrid adsorbent was prepared by the sol–gel process in an acidic medium. First, 0.3 mL of EtOH, 0.1 mL of distilled water, and 0.3 mL of concentrated HCl were added to 0.4 mL of TEOS. The mixture was stirred vigorously for 10 min at 60 °C and 420 rpm to obtain a uniform solution. To prepare a 1:1 ratio of inulin and TEOS, 0.8 mL of inulin solution was slowly added dropwise into the mixture solution. The mixture was then allowed to react for 2 h. The same steps were repeated to obtain a 1:2 ratio of inulin and TEOS by varying the molar composition of EtOH and HCl (0.6 mL), H_2_O (0.2 mL), and TEOS (0.8 mL). Meanwhile, 1.6 mL inulin solution with constant TEOS solution was used to prepare the adsorbent at a 2:1 ratio. The prepared sol–gel inulin–silica adsorbents were dried in an oven at various temperatures (50, 60, and 70 °C) for 1 h to remove the excess water content. The adsorbents were stored in zip-locked plastic bags at room temperature before undergoing physical, structural, and morphological characterization.

FTIR analysis was conducted to characterize the functional group of the hybrid adsorbents using a PerkinElmer spectrophotometer. The potassium bromide (KBr) method was used to analyze inulin powder and inulin–TEOS composite at a range of 4000 to 400 cm^–1^. An attenuated total reflectance (ATR) method commonly used for liquid and solid materials was used to analyze raw TEOS at range of 4000 to 700 cm^–1^. Before further characterization, the adsorption performance of heavy metals was compared for different mol ratios of inulin–TEOS hybrid adsorbent (1:1, 2:1, and 1:2). For this, a 1.0 ppm solution of lead, nickel, cobalt, and cadmium was prepared, then 0.1 g of each adsorbent was added to 10 mL of heavy metal solution and agitated for 15 min on a rotary shaker. Then, the adsorbent was separated from the solution using a 0.45 μm polypropylene syringe filter, and the residual concentration of heavy metal was measured by ICP-MS. The percentage removal of heavy metal was calculated using the following equation:

R (%) = [(C_o_ − C_e_)/C_o_] × 100

where C_o_ is the initial concentration and C_e_ is the measured concentration of heavy metal in the solution (ppm) after adsorption.

The morphology of inulin–TEOS adsorbent was observed by a JSM-7800F field emission scanning electron microscope. The elemental composition and weight percentage of elements was analyzed by EDX using an Oxford TM3030Plus microscope with acquisition conditions of 30 s and 15 kV of accelerating voltage. A Rigaku Miniflex XRD system with CuK α radiation (λ = 1.54 Å) within the range of 10–80° with 2° per min scanning speed was used to determine the crystalline structure of inulin–TEOS adsorbent. BET analysis was applied to discover the specific surface area, pore volume, and pore size of the adsorbent through a Micromeritics ASAP 2020 analyzer. TGA was performed on a thermoanalyzer (Q 500). In each case, samples were examined under nitrogen atmosphere with flow rate of 50 mL/min and heating rate of 10 °C/min from 30 to 900 °C. The static contact angle of 2:1 inulin–TEOS hybrid adsorbent was measured by an OCA40 contact angle system (Dataphysics, Germany) using a 5 μL water droplet at ambient temperature.

## 3. Results and Discussion

### 3.1. Synthesis of Inulin–TEOS Adsorbent

The formation of inulin–TEOS hybrid by sol–gel undergoes a two-network forming process. The initial reaction is the catalytic hydrolysis of TEOS in the presence of water and HCl. The alkoxylated agent TEOS was used due to its affinity with water [15]. However, TEOS and water are immiscible, and thus ethanol, which acts as a homogenizing agent, was used to facilitate the hydrolysis process [16]. The presence of ethanol results in miscibility of alkoxide and water, which form a homogeneous solution [17,18]. HCl was added to the solution to shorten the hydrolysis time and hasten the gelation of the sol, since Si(OR)_4_ reacts slowly in water and alcohol.

The polycondensation reaction of hydrolyzed TEOS occurred after the hydrolysis reaction and produced a longer polymer network [19]. Under acidic conditions, the condensation reaction, which involves protonated silanol species, makes the silicon more electrophilic and thus more prone to nucleophilic attack. Hence, this condition induces the condensation reaction that occurs between neutral species and protonated silanol situated at the end groups of chains, resulting in the creation of siloxane networks [17].

**Scheme 1:**
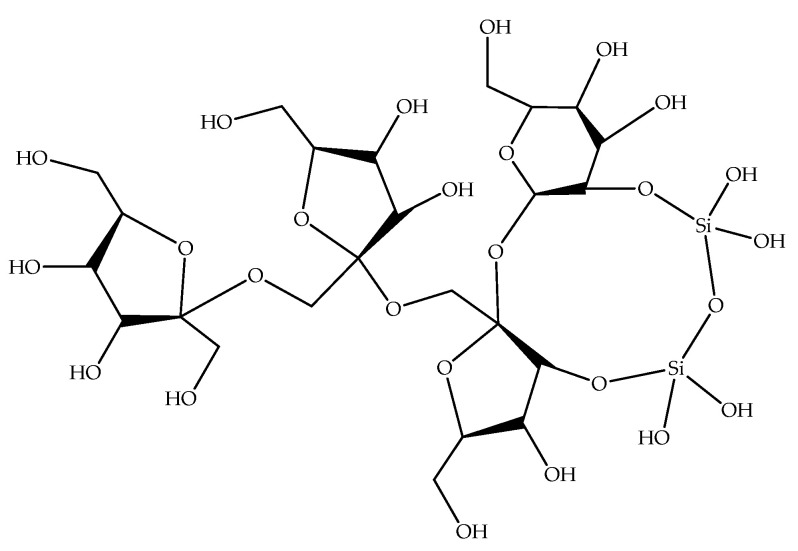
Inulin–tetraethoxysilane (TEOS) hybrid.

The last step is the bonding process between inulin and the silanol group. The polyhydroxy compounds of inulin consist of numerous monosaccharide residues at their hydroxyl groups, resulting in silica nucleation due to the hydrolysis of TEOS. Also, the hydrolysis of TEOS, the condensation of created silanol groups, and the reaction of silanol groups of TEOS with carbonyl groups of inulin generates Si–O–C bonds [20]. Alkoxysilane also incorporates into the matrix via hydrogen bonding and does not form covalent bonds with amine groups, but acts as an excellent hydrogen-bonding partner and facilitates the formation of networks (Scheme 1).

### 3.2. Characterization

**Figure 1:**
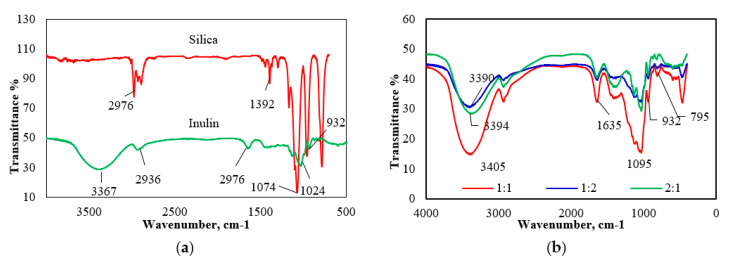
Comparison of FTIR spectra: (**a**) raw inulin and TEOS; (**b**) inulin–TEOS hybrid with different molar ratios.

The raw inulin, TEOS, and inulin–TEOS composite with different molar ratios were characterized using FTIR. The raw inulin and TEOS were compared with the hybrid inulin–TEOS spectrum (Figure 1). The characteristic CH_3_ and CH_2_ bands in the ethoxy groups comprising the TEOS in the range of 3000 cm^–1^ nearly disappeared in the spectrum of hybrid samples. This is due to the evaporation of ethanol, which causes the removal of terminal Si-OH groups during the reaction process. Several bands were observed at 3438, 3394, and 3390 cm^–1^ for 1:1, 1:2, and 2:1 composites, respectively, corresponding to O-H stretching vibrations and H-O-H bending vibrations generated by water [21]. The formation of hydrogen bonds between the silanol group of the silica network and the hydroxyl group of inulin results in the appearance of broad and strong bands in the spectra [22]. The Si-O-Si network was allocated to the region of 1000–1250 cm^–1^. The band located at 932 cm^–1^ was attributed to Si-OH stretching, which corresponds to polymerization between inulin and hydrolyzed TEOS. The large intensity of the peak at 1095 cm^–1^ was due mainly to the overlapping of Si-O-Si and the –C-O-C– of ring ether [23,24]. The presence of Si-O-C networks in inulin–TEOS at 1061 and 795 cm^–1^ confirms the interaction between inulin and TEOS. The significant characteristic absorption bands for raw inulin were found to be shifted from 1644 cm^–1^ (C=O stretching from carbonyl band, indicating possible acetylation) to 1635 cm^–1^ in the hybrid material.

**Figure 2:**
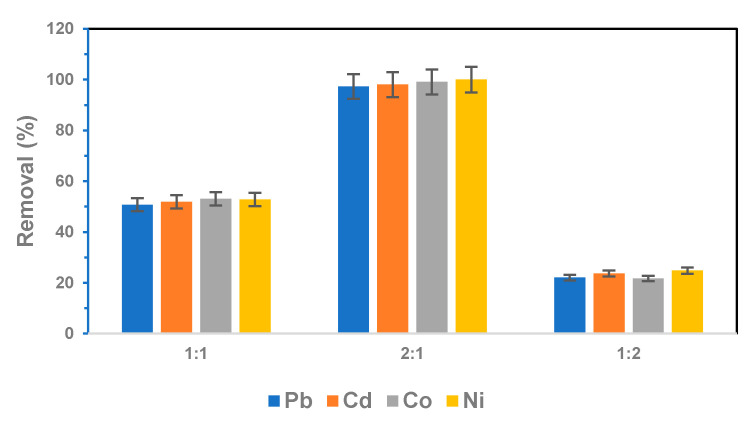
Comparison of adsorption performance of 1:1, 2:1, and 1:2 inulin–TEOS hybrid adsorbent with different metal ions.

The inulin–TEOS hybrid sample with 2:1 molar ratio dried at 70 °C with a weight of 0.17 g was chosen for further characterization, due to its adsorption performance. The adsorption performance of three molar ratios of inulin–TEOS hybrid adsorbents was investigated to remove various heavy metal ions. The results are plotted in Figure 2. The percentage removal of the 2:1 inulin–TEOS hybrid adsorbent for all heavy metals was 2 to 5 times higher than that of adsorbents with 1:1 and 1:2 molar ratio. It was found that a higher concentration of inulin provides a higher percentage of cation removal. This can be explained by the high quantity of hydroxyl groups of inulin, which is capable of heavy metal ion bonding [25].

**Figure 3:**
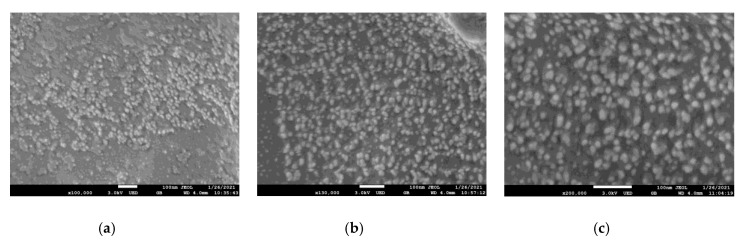
FESEM micrographs of 2:1 molar ratio inulin–TEOS at (**a**) 100 k, (**b**) 130 k, and (**c**) 200 k magnification.

The morphology of inulin–TEOS hybrid with 2:1 molar ratio observed by FESEM under different magnifications is shown in Figure 3. The surface of the hybrid seems to be rough, and it consists of nanoparticles with a size of 14–21 nm. The presence of irregular pores is due to the presence of TEOS, which has the irregularly spaced and broadly distributed pores of different sizes [26]. The aggregation of these nanoparticles results in mesoporous formation in the hybrid materials [27]. The well-defined mesoporous structure was further confirmed by BET analysis.

**Figure 4:**
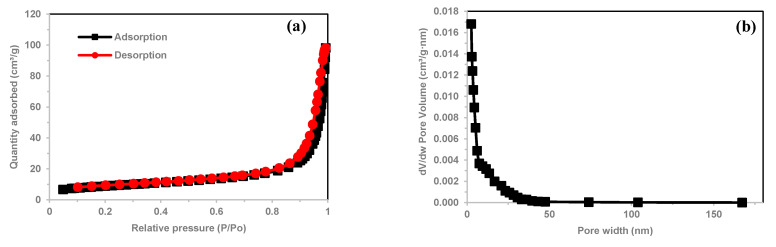
(**a**) N_2_ adsorption/desorption isotherm and (**b**) pore size distribution curve of 2:1 inulin–TEOS hybrid adsorbent.

The surface area, pore volume, and pore size of 2:1 molar ratio inulin–TEOS were determined in nitrogen adsorption and desorption environments using the BET method. The BET surface area for the inulin–TEOS hybrid was 17.69 m^2^/g. The pore size obtained was between 2 and 50 nm (mesoporous), with pore volume of 0.02 cm^3^/g (Figure 4). Adsorption depends on the area, because it is a surface phenomenon. Very little adsorption will occur in bigger pores compared to smaller pores. This is because big pores have less exposed surface, which directly decreases the absorbance capacity. In other words, big pores have a low area-to-volume ratio [28]. Nevertheless, bigger pores could play a role in increasing the diffusion of targeted pollutants.

**Figure 5:**
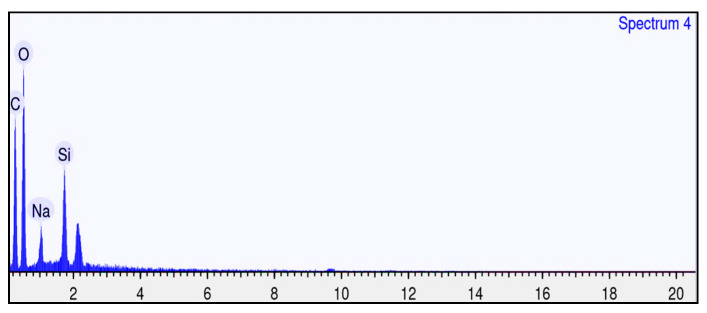
EDX spectra for 2:1 molar ratio inulin–TEOS hybrid adsorbent.

Energy-dispersive X-ray was carried out to identify the elemental composition (Figure 5) and weight percentage (Table 1) of elements present in the adsorbent. It was also used to verify the functional groups observed by FTIR, which proved the successful interaction between inulin and TEOS. As expected, EDX analysis confirmed the presence of C, O, and Si elements in the hybrid sample. The existence of Si atoms corresponds to the silicon alkoxide precursor due to the use of TEOS in sol–gel synthesis. Na element was also present in the hybrid sample due to the use of sodium hydroxide (NaOH) as solvent to dissolve inulin. The high-intensity peak of C and O elements suggests that the hybrid composite consisted predominantly of carbon and oxygen atoms obtained from the organic compound. According to Bello et al. [29], material rich in carbon content has efficient adsorbent properties for the removal of heavy metals, dyes, and other organic pollutants from aqueous solution due to high surface area and production of mesopores.

**Figure 6:**
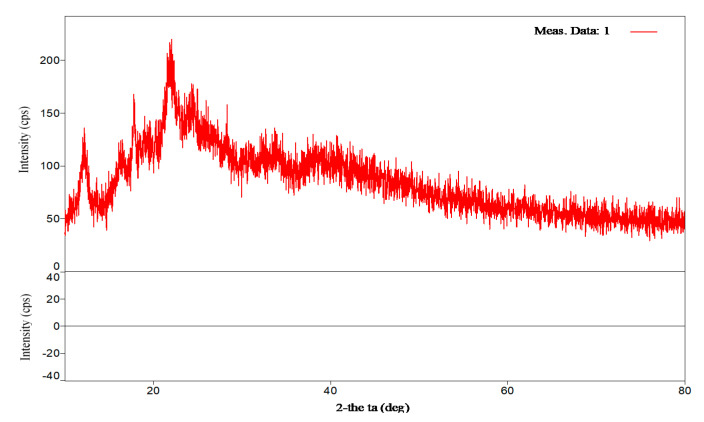
XRD pattern of 2:1 molar ratio inulin–TEOS adsorbent.

The phase composition of the synthesized inulin–TEOS hybrid adsorbent was analyzed by XRD (Figure 6). The most significant peak was observed at 22° in the 2θ angle, which shows the amorphous state of the silica network [30,31]. According to Ronkart et al., inulin could form a semi-crystalline structure in the presence of water, which was represented at peaks around 12°, 16.4°, 17.7°, 21.4°, 23.8°, and 37.37° [32]. The tridymite structure formed at 12.05° in current study was influenced by inulin. Thus, it can be suggested that inulin exists as an anhydrous crystalline phase. The results indicate that the 2:1 molar ratio inulin–TEOS hybrid was in amorphous and semicrystalline form. These structures influenced the surface area and porosity of the adsorbent and directly affected the adsorption capacity [33,34].

**Figure 7:**
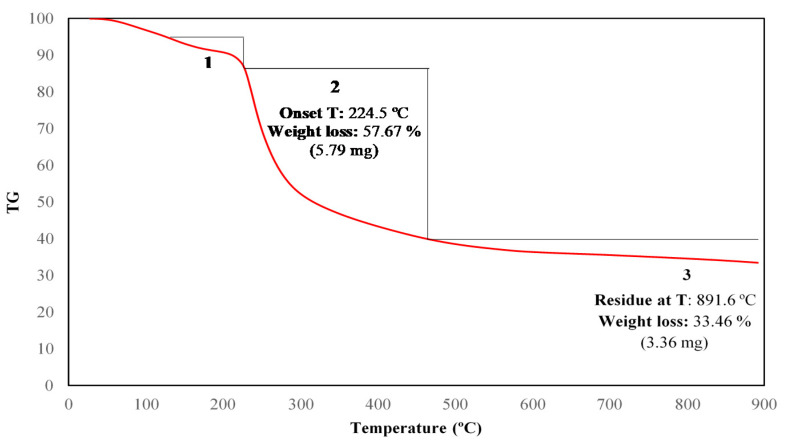
Thermogravimetric profile of 2:1 molar ratio inulin–TEOS hybrid adsorbent.

**Figure 8:**
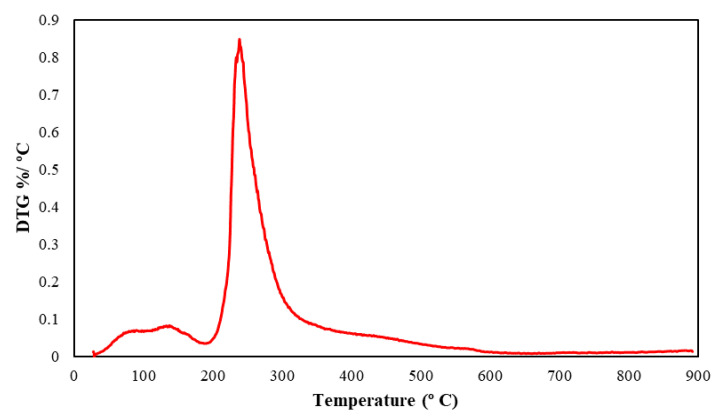
Derivative thermogravimetric profile of 2:1 molar ratio inulin–TEOS hybrid adsorbent.

The thermal stability of 2:1 molar ratio inulin–TEOS hybrid was investigated by TGA (Figure 7). The TGA profile clearly indicates that the degradation occurred in the range between 120 and 180 °C (1). This is due to water desorption from the composite surface and the side product of subsequent condensation of the Si–OH groups [22]. In addition, physicochemical transformations such as dehydration, melting, changes in conformation of molecules, initial defragmentation, etc., occurred at low temperature [35]. The more significant thermal decomposition was observed at temperatures between 180 and 450 °C (2) with extensive weight loss of about 57.76%, which was most likely caused by dehydration of the saccharide rings, depolymerization, and decomposition of organic groups [36]. The degradation process was completed at a temperature of 800 °C (3), when the hybrid remained thermally stable. Based on derivative thermogravimetric (DTG) peaks (Figure 8), the degradation occurred in the form of exothermic peaks, confirming that the stages of weight loss were caused by oxidative degradation [6].

**Figure 9:**
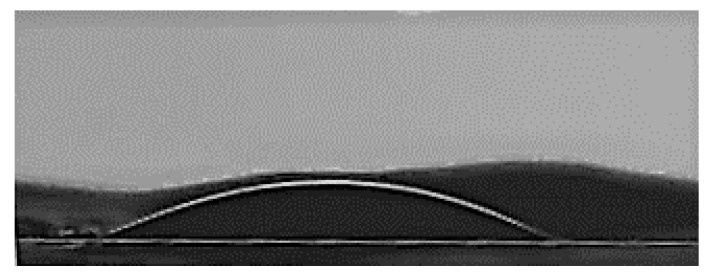
Water contact angle on inulin–TEOS hybrid adsorbent.

The static contact angle was measured on the 2:1 inulin–TEOS hybrid adsorbent using the sessile drop method. The contact angle was 31.1°, as shown in Figure 9, indicating that the adsorbent exhibited hydrophilicity, which is quite beneficial for adsorbents in an aqueous system [37].

## 4. Conclusions

In this research, a new inulin–TEOS hybrid for use as an adsorbent was successfully synthesized via the sol–gel method under acidic conditions. The molar ratio of inulin to TEOS and the drying temperature were found to affect the interaction between the two substances. A molar ratio of 2:1 and a drying temperature of 70 °C were selected as the best parameters for the inulin–TEOS hybrid. The hybrid showed the interaction between inulin and TEOS by bands located at 1061 and 795 cm^–1^, which indicated the formation of Si–O–C network. The appearance of a yellowish crystal powder confirmed the presence of inulin in the composite. In addition, the XRD pattern confirmed that the composite was in an amorphous and semi-crystal form, verified by the presence of a rough and irregular surface as depicted by FESEM. The mesoporous structure of the adsorbent revealed by BET isotherm indicates that inulin–TEOS is a promising adsorbent. According to TGA, the inulin–TEOS adsorbent was thermally stable under 200 °C. Based on these characteristics, inulin may offer great potential as a low-cost adsorbent.

## Figures and Tables

**Table 1:** Quantity of elemental composition in 2:1 molar ratio inulin–TEOS adsorbent.

Element	Weight %	Atomic %
Carbon	40.122	48.490
Oxygen	51.818	47.016
Silicon	5.193	2.684
Sodium	2.867	1.810

## Data Availability

The data presented in this study are available in the paper.
